# Evaluation of effectiveness and analysis of goal-directed blood transfusion in peri-operation of major orthopedic surgery in elderly patients

**DOI:** 10.3892/etm.2012.834

**Published:** 2012-11-26

**Authors:** HONG ZHENG, JIAN-JIANG WU, JIANG WANG

**Affiliations:** Department of Anesthesiology, The First Affiliated Hospital of Xinjiang Medical University, Urumqi 830054, P.R. China

**Keywords:** orthopedic elderly patients, goal orientation, blood transfusion, effectiveness

## Abstract

Through the establishment of blood transfusion-effectiveness assessment criteria, we aimed to evaluate and analyze the effectiveness of peri-operative red blood cell transfusion for major orthopedics in elderly patients. Male and female patients (n=106) with American Society of Anesthesiologists (ASA) stage II–III, aged 60–80 years and scheduled for elective orthopedic surgery were randomly divided into 2 groups: group I (n=52), received the traditional method of red blood cell transfusion and group II (n=54) received hemoglobin (Hb) and hematocrit (Hct) goal-directed red blood cell (RBC) transfusion. We compared the changes in Hb, Hct, RBC, platelet (PLT), prothrombin time (PT), activated partial thromboplastin time (APTT), alveolar-arterial oxygen partial pressure [P(A-a)O_2_], oxygenation index (OI) and Vigileo monitoring indicators following RBC composition transfusion before and after surgery. We also monitored wound healing time, number of hospitalization days and intensive care unit (ICU) transfer rate. Blood transfusion-effectiveness assessment criteria were used to evaluate the efficacy and safety of Hb and Hct goal-directed RBC transfusion in orthopedic elderly patients. The two groups demonstrated an efficiency of 61.5 and 72.2%, respectively (P>0.05). The P(A-a)O_2_ and OI in the two groups were not significantly different (P>0.05). Compared with the traditional RBC transfusion group, the Hb and Hct goal-directed RBC transfusion group presented increased RBC, Hb and Hct, as well as decreased PT and APTT and shorter wound healing time. The number of days of hospitalization and ICU transfer rate also decreased (P<0.05). The Hb and Hct goal-directed method for RBC transfusion was found to be more effective as a form of peri-operative RBC transfusion for major orthopedics in elderly patients.

## Introduction

Blood protection in major orthopedic surgery has become a focus of attention and research, particularly with prolonged life expectancy, the development of orthopedic surgery technology and the constantly enhanced blood conservation awareness. In major orthopedic surgery, the proportion of elderly patients increases each year (1). Due to the decline in organ function, elderly patients have a significantly increased postoperative infection rate and mortality compared with young and middle-aged patients under the same condition of blood loss (2,3). Significant blood loss is one cause of peri-operative complications in elderly patients (4,5). A method to evaluate the effectiveness of blood transfusions is a current key issue. However, in all peri-operative blood transfusion guides, only the circumstances in which blood transfusions are needed is mentioned, not how to correctly and reasonably give a blood transfusion to elicit the best effect. Therefore, an evaluation system of blood transfusion effectiveness is needed. In this study, we compared the traditional red blood cell (RBC) trans-fusion method to the hemoglobin (Hb) and hematocrit (Hct) goal-directed RBC transfusion method to objectively evaluate the effectiveness of blood transfusion in peri-operation of major surgery in elderly patients, thereby providing theoretical basis to establish reasonable and individual clinical blood conservation strategies.

## Materials and methods

### Subjects

This study was approved by the Ethics Committee of the First Affiliated Hospital of Xinjiang Medical University and patients provided informed consent. A total of 106 male and female patients were included from orthopedic elective surgeries. Patients were classified as American Society of Anesthesiologists (ASA) stage II–III, according to their systemic conditions. The average age was 70±6 years and the average weight was 68±13 kg. Eight patients had a history of more than two blood transfusions. Patients were randomly divided into the control group (n=52) and the experimental group (n=54) using the envelope method. The control group (group 1) received the traditional RBC transfusion method and the experimental group (group 2) received the Hb and Hct goal-directed RBC transfusion method.

The inclusion criteria were as follows: according to the World Health Organisation (WHO) criteria, patients were aged 60–80 years, with cardiac function ≤ level II and did not have a preoperative history of mental or neurological disease or transfusion allergy. The expected intraoperative blood loss was >20% of the total blood volume. Patients were required to be in accordance with the transfusion indications of RBC suspension stipulated in the Clinical transfusion technical specifications (2000) by the Chinese Ministry of Health.

The exclusion criteria were as follows: above ASA level III, temperature >38°C, a history of coronary heart disease or diabetes, ejection fraction (EF) <50%, serious liver or kidney dysfunction, long-term use of anticoagulant drugs, severe coagulation disorders, severe chronic obstructive or restrictive pulmonary disease, long-term use of β-retardants, a history of central nervous system diseases, malnutrition with low weight or obese patients, severe peripheral vascular occlusive disease, intraoperative and postoperative use of erythropoietin (EPO) (6) and receiving hormone therapy. No other factors were considered relevant for the present study.

### Anaesthesia methods

The patients were not administered preoperative premedication; however, they were provided with oxygen masks and monitoring electrocardiogram (ECG), heart rate (HR), blood pressure (BP) and pulse oxygen saturation (SpO_2_) after entering the operating room. The peripheral pathway was established by infusing 0.9% sodium chloride injection for 10 ml/kg/h through the left upper limb vein. The left radial artery was punctured for catheterization and connected to an invasive blood pressure monitor and patients were given an oxygen inhalation face mask at 3 l/min. The anaesthetic induction was an intravenous injection of 0.5–1.0 μg/kg sufentanil, 0.1–0.15 mg/kg midazolam, 0.1 mg/kg vecuronium bromide and 0.5–1.0 mg/kg isopropylphenol as well as orotracheal intubation and mechanical ventilation after 5 min. Ventilation parameters were 8–10 ml/kg tidal volume, 10–12 times/min respiratory rate and sustained respiratory end-tidal carbon dioxide (PETCO_2_; 35–40 mmHg). Anesthesia was maintained by continuous intravenous infusion of 4–6 mg/kg/h isopropylphenol and 0.05–0.1 mg/kg/h vecuronium bromide and a simultaneous discontinuous inhalation of 1–1.5% sevoflurane. The non-treatment factors were controlled by the use of air conditioning and electric blankets to keep the nasopharyngeal temperature at 36.5–37.2°C. The capacity was controlled and 0.9% sodium chloride injection was infused for 10 ml/kg/h before blood transfusion to maintain a stable circulation system. To optimize tissue perfusion adequate colloid or crystalloid fluid was infused in elderly patients. The peri-operative 12-lead ECG was monitored to prevent myocardial ischemia and myocardial infarction. Blood loss was quantified in strict accordance with the standard method.

### Evaluation method of transfusion

The Hb within 12 h of transfusion with a RBC suspension was compared to Hb prior to blood transfusion to calculate the increment. In accordance with the traditional evaluation method, we estimated that the effective infusion of RBCs was ∼5 g/l Hb increased in every 1 unit transfused RBC suspension or ∼10 g/l Hb increased in every 3 units transfused washed RBCs.

For the Hb and Hct goal-directed RBC transfusion method, a portable i-STAT 1 blood gas analyzer was employed to record peri-operative Hb and Hct at varying time points to dynamically monitor the most appropriate time for RBC transfusion, according to the infusion indications of RBC suspension stipulated in the Clinical transfusion technical specifications (2000) by the Chinese Ministry of Health.

As yet there is no evaluation of the effectiveness of blood transfusion in peri-operation of major orthopedic surgery in elderly patients. Older patients with hemorrhage as well as significant transfusion are at risk of postoperative mortality (7). The purpose of blood transfusion is to maintain the balance of oxygen supply and oxygen consumption. The oxygenation index (OI) reflects the balance between body oxygen supply and oxygen consumption, and the change in Hb and Hct reflects the improved oxygen-carrying capacity prior to and after blood transfusion. OI and Hb as well as cardiac output (CO) are positively correlated. Cardiac output is reflected by hemodynamic index, heart rate and blood pressure. Therefore, we maintained an intraoperative balance of body oxygen supply and oxygen consumption and produced a standard for evaluating blood transfusion effectiveness through accurate physiological calculation. Scoring was based on indices that are easily monitored, including Hb, Hct, activated partial thromboplastin time (APTT), prothrombin time (PT), OI, blood transfusion complications, wound healing time, length of stay and the number of transfers to the intensive care unit (ICU). As a result, the Hb and Hct goal-directed RBC transfusion method was compared to the traditional blood transfusion method in order to establish a standard for evaluation of blood transfusion effectiveness to guide blood transfusion in the peri-operation of major orthopedic surgery in elderly patients.

The evaluation of the ascensional range of Hb, Hct, PT, APTT and OI following transfusion of RBC suspension is shown in Table I. The evaluation of the adverse transfusion reactions, as well as complication rate, wound healing time, length of stay and ICU transfer rate is shown in Table II. The transfusion reactions included anaphylactic reactions or anaphylactoid reactions, febrile reactions, electrolytes, acid-base imbalance, citrate poisoning, hypothermia caused by massive transfusion of cold blood, phlebitis after transfusion, transfusion-related lung injury, hemolytic transfusion reaction and transfusion-related infections.

The standard for evaluation of blood transfusion effectiveness was: <5, invalid; >8, valid and >10, highly effective (Tables I and II).

### Monitoring indicators

The general data of patients were recorded. Patients were given radial arterial blood gas analysis using a portable blood gas analyzer (i-STAT 1, Abbott Laboratories, IL, USA) after entering the operating room and Hb, Hct and RBC were recorded. The heart rate (HR), blood pressure (BP), temperature (T) and SpO_2_ were recorded with a S/5 Avance anesthesia machine (Datex Ohmeda, Finland) and a BeneviewT6 patient monitor (Shenzhen Mindray Bio-Medical Electronics Co., Ltd., China). At the same time, the cardiac index (CI), stroke volume index (SVI) and the corresponding systolic blood pressure (SBP), diastolic blood pressure (DBP), mean arterial pressure (MAP) and heart rate (HR) were monitored with a Flotrac sensor and Vigileo monitor (software version 1.07, Edwards Lifesciences, Irvine, CA, USA) to calculate the surrounding vascular resistance (SVR = MAP x 80/CO) as a basic value (T0). Thereafter, the radial arterial blood gas analysis was performed at 12 h (T1), 24 h (T2) and 48 h (T3) after blood transfusion. At the same time, surgery time (from skin incision to the end of surgery), adverse transfusion reactions, complication rate, wound healing time, length of stay, ICU transfer rate and intraoperative RBC transfer volumes were recorded.

The main monitoring indicators included Hb and Hct and the secondary monitoring indicators included APTT, PT, P(A-a)O_2_, OI and Vigileo monitored indicators. Increased P(A-a)O_2_ caused by pathological factors, including anatomical shunt, ventilation/perfusion imbalance and impeded diffusion of alveolar-capillary barriers was ruled out (8). The computational formula for P(A-a)O_2_ was as follows: P(A-a)O_2_ = [(atmospheric pressure - water vapor pressure x fraction of inspired oxygen (FiO_2_) - PaCO_2_/R] - PaO_2_, where the atmospheric pressure was 760 mmHg; the water vapor pressure, 47 mmHg and the respiratory quotient (R), 0.8. PaO_2_ and PaCO_2_ were measured by arterial blood gas. The formula for OI was as follows: OI = PaO_2_/FiO_2_.

The outcome indicators that were monitored included adverse transfusion reactions and complication rate, wound healing time, length of stay and ICU transfer rate.

Intraoperative blood loss was monitored and we calculated the observation time in the surgical field and communicated it with the surgeon to evaluate whether there was a significant amount of microvascular bleeding. Standard methods were taken to quantify the blood loss, including suction apparatus, gauze measurement and recycled blood volume. The formula for RBC suspension supplementation was: suspension supplementation of RBCs = [(predicted Hct - practical Hct) x 55 x weight]/0.60.

### Statistical analysis

Patients were randomly divided into different groups by the envelope method. Data were analyzed by SPSS 13.0 statistical software (SPSS Inc., Chicago, IL, USA). Measurement data were expressed as mean ± standard deviation and enumeration data were determined by the univariate χ^2^ test. Comparison among groups was performed using a t-test and comparison within groups was performed using the variance analysis of repeated measurement data. P<0.05 was considered to indicate a statistically significant difference.

## Results

### General data

Intraoperative transfusion of a RBC suspension in 106 patients were all >2 units (400 ml) and there were no intraoperative or postoperative transfusion-related allergic reactions or other related complications. There were 3 patients in group 1 that were transferred to the ICU for further treatment, with a transfer rate of 5.6% and an ICU detention time >24 h. There were no patients in group 2 that were transferred to the ICU. There was no statistical difference in age, height, weight, body surface area, cardiac function, duration of surgery, intraoperative transfusion volume of RBCs and length of stay between the two groups (P>0.05; Table III).

### Hb and Hct

The comparison of parameters before and after blood transfusion in the two groups (Table II) revealed a significant difference in the Hb and Hct goal-directed RBC transfusion method (P>0.05). There was a statistical difference in RBC and PT before and after blood transfusion in the traditional method of RBC transfusion (P<0.05; Table IV). Our results indicate that the changes in Hb, Hct, RBC, platelet (PLT), PT and APTT before and after blood transfusion are greater in the Hb and Hct goal-directed RBC transfusion method compared with the traditional method.

### P(A-a)O_2_ difference

There were no significant differences in P(A-a)O_2_ and OI preoperatively (T0), at 12 h (T1), 24 h (T2) or 48 h (T3) after blood transfusion between the two groups (P>0.05; Table V), indicating that there is no difference in P(A-a)O_2_ change and OI before and after transfusion between the two groups.

### RBC transfusion

Referring to the transfusion indications of RBC suspension stipulated in the Clinical transfusion technical specifications by the Chinese Ministry of Health, Hb should be 70–100 g/l, which is determined according to the degree of anemia, cardiopulmonary compensatory function, age and whether there is an increased metabolic rate, as well as other factors. In this study, compared to the traditional RBC transfusion method, we noted that in the Hb and Hct goal-directed RBC transfusion method, SpO_2_, body temperature and HR fluctuated when Hb <10 g/dl. Therefore, Hb <10 g/dl is considered as one of the standards of RBC transfusion in this group (9).

The rate of effectiveness in the traditional and Hb and Hct goal-directed RBC transfusion methods were 61.5 and 72.2%, respectively. The rate of effectiveness of the Hb and Hct goal-directed RBC transfusion method is clearly higher than that of the traditional transfusion method.

## Discussion

Our study has demonstrated that compared with the traditional method of RBC transfusion, the Hb and Hct goal-directed RBC transfusion method clearly reduces the ratio of invalid RBCs infused, with a blood transfusion inefficiency of 38.5 and 27.8% respectively. This was due to not excluding ongoing blood loss, blood infiltration outside the blood vessel, fluid hemodilution and RBC loss in the extracorporeal circulation, as well as other factors.

Comparing different parameters before and after blood transfusion in the two groups and at the same time referring to the scoring method of blood transfusion effectiveness, we noted that compared to the group with traditional transfusion, the RBC, Hb and Hct in the Hb and Hct goal-directed RBC transfusion patients increased, APTT and PT decreased, wound healing time and hospitalization days shortened and ICU transfer rate reduced (P<0.05). There was no statistical difference in P(A-a)O_2_ changes or OI between the two groups in the peri-operative period (P>0.05). The reason for this may be related to normal or compensatory cardiopulmonary function in orthopedic elderly patients. At the same time, intraoperative RBC suspension volumes in 106 patients were all >2 units (400 ml) and there were no intraoperative and postoperative allergic reactions or other complications related to blood transfusion. The wound healing time and number of hospitalization days in group 1 were significantly higher than those in group 2. Three patients in group 1 were transferred into the ICU for further treatment, with a transfer rate of 5.6% and ICU detention time was >24 h. No patients were transferred into the ICU from group 2.

This study aimed to establish a clinical scoring method to correctly evaluate the effectiveness of blood transfusion to achieve scientific, reasonable and effective blood transfusion. A number of studies have evaluated the effectiveness of RBC transfusion (10,11) with regard to whether Hb achieved the expected value after blood transfusion; however, there has been no evaluation of whether the clinical symptoms of the patients improved or monitoring of complications following blood transfusion (12). Low Hb in surgery patients may increase the incidence rate of postoperative complications (13,14), including the extension of postoperative recovery time (15,16) and an increase in postoperative infection susceptibility (17,18). Analysis of patients with postoperative Hb <8 g/dl revealed that the mortality rate increased for postoperative Hb <7 g/dl (19). When Hb was <6 g/dl, memory changes appeared (20) and when Hb was <5 g/dl, the mortality significantly increased (21,22). In this study, the blood transfusion strategy using Hb and Hct goal-direction may not only effectively save 10.7% blood, but also significantly reduce the wound healing time, number of hospitalization days and ICU transfer rate to significantly improve patient outcome and prognosis.

The limitations of this study are that it was a randomized control study in a single center with a small sample size and the objects of the study were older patients undergoing orthopedic surgery. Therefore, further investigation is required to determine whether this evaluation method of blood transfusion effectiveness is also applicable to other patients with different types or ages.

The scoring method of blood transfusion effectiveness was established through comparing the Hb and Hct goal-directed RBC transfusion method to the traditional RBC transfusion method. Our findings indicated that the Hb and Hct goal-directed RBC transfusion method improves the blood transfusion effectiveness in the peri-operative period of major orthopedic surgery in elderly patients. At the same time we identified that Hb <10 g/dl, as one of the blood transfusion standards in orthopedic elderly patients, improves the blood transfusion effectiveness in the peri-operative period of major orthopedic surgery in elderly patients. The ineffectiveness of RBC transfusion is mainly related to postoperative blood loss, the isoimmunization of multiple transfusions and accelerated destruction, as well as other factors. Therefore, minimizing the number of blood transfusions, as well as controlling infection and fever may improve the effectiveness of RBC transfusion treatment.

## Figures and Tables

**Table I.** Blood transfusion effectiveness score.

Scores	Hb	Hct	PT	APTT	OI (mmHg)
5	Normal	Normal	Normal	Normal	>300
+1	Increased 1 g/dl	Increased 1%	−1 sec	−3 sec	300–400
+2	Increased 2 g/dl	Increased 2%	−2 sec	−6 sec	>400

Hb, hemoglobin; Hct, hematocrit; PT, prothrombin time; APTT, activated partial thromboplastin time; OI, oxygenation index.

**Table II.** Blood transfusion effectiveness score.

Scores	Transfusion complications	Wound healing time (days)	Length of stay (days)	ICU transfer rate
5	None	<12	<10	None
−1	1 item	12–15	10–12	1 time
−2	2 items	>15	>12	≥2 times

ICU, intensive care unit.

**Table III.** Comparison of general data in patients.

Parameters	Control group	Experimental group	P-value (χ^2^)
Age (years)	69.2±5.9	70.1±5.8	0.974
Gender (n)			
Male	24	21	0.449 (0.572)
Female	28	33	
Height (cm)	174.2±7.8	174.3±4.9	0.941
Weight (kg)	80.1±18.1	76.2±10.1	0.370
Body surface area (m^2^)	2.05±0.26	2.00±0.15	0.452
Cardiac function			
Grade 1	12	15	0.890 (1.270)
Grade 2	40	39	
Duration of surgery (min)	166.0±66.7	194.6±82.4	0.051
Intraoperative red blood cells (unit)	2.4±1.2	2.7±1.4	0.300
Length of stay (days)	20.0±10.0	22.0±10.0	0.371

The general conditions of patients in the experimental group were compared to those in the control group (P>0.05, Chi-square test). Data are presented as the mean ± standard deviation.

**Table IV.** Comparison of parameters before and after transfusion.

Parameters	Group	Before transfusion	After transfusion	P-value
RBC (×10^12^/l)	I	4.0±0.8	3.8±0.6	0.033
	II	4.1±0.6	3.7±0.6	0.003
PLT (×10^9^/l)	I	209.4±79.1	213.1±75.8	0.746
	II	181.1±60.1	166.5±59.0	0.002
Hct (%)	I	38.9±5.1	38.0±5.0	0.287
	II	37.5±6.3	34.5±5.6	0.006
Hb (g/l)	I	118.7±19.7	113.4±17.0	0.161
	II	126.0±21.1	119.3±22.5	0.007
PT (sec)	I	7.1±2.0	8.0±2.0	0.032
	II	8.3±3.0	7.2±3.0	0.024
APTT (sec)	I	24.0±6.0	25.0±5.0	0.057
	II	23.0±4.0	25.0±3.0	0.016

Data are presented as the mean ± standard deviation. I, control group; II, experimental group; RBC, red blood cell; PLT, platelet; Hct, hematocrit; Hb, hemoglobin; PT, prothrombin time; APTT, activated partial thromboplastin time.

**Table V.** Alveolar-arterial oxygen partial pressure difference, oxygenation index and Vigileo monitored indicators at different time points in the two groups.

Monitoring indicators	Time point	Group I	Group II
P(A-a)O_2_ (mmHg)	T0	21.3±5.2	20.9±4.9
	T1	343.7±110.2	374.2±123.1
	T2	418.5±95.3	417.4±137.3
	T3	208.2±104.4	181.2±89.1
OI	T0	379.4±32.3	400.0±23.7
	T1	322.7±105.0	290.2±123.4
	T2	239.2±95.1	229.6±166.8
	T3	227.2±98.2	243.9±119.3
MAP (mmHg)	T0	94±17	97±15
	T1	81±19	80±13
	T2	76±14	68±13
	T3	81±10	80±7
HR (bpm)	T0	75±13	67±21
	T1	74±15	69±13
	T2	78±14	67±13^a^
	T3	95±15	109±15^a^
CI (l/min/m^2^)	T0	3.1±0.7	3.6±0.8
	T1	2.6±0.7	2.8±0.6
	T2	2.8±0.6	2.5±0.4^a^
	T3	3.5±0.8	3.3±0.7
SVI (ml/beat/m^2^)	T0	41±10	46±13
	T1	37±10	41±7
	T2	37±7	40±8
	T3	38±6	33±6^a^
SVR (dynes-sec/cm^5^)	T0	1375±258	1243±318
	T1	2525±535	2362±359
	T2	1247±264	1258±340
	T3	1058±288	1110±258

a P<0.05 vs group 1. Data are presented as the mean ± standard deviation. P(A-a)O_2_, alveolar-arterial oxygen partial pressure; OI, oxygenation index; MAP, mean arterial pressure; HR, heart rate; CI, cardiac index; SVI, stroke volume index; SVR, surrounding vascular resistance.

## References

[1] Leung JM, Dzankic S (2001). Relative importance of preoperative health status versus intraoperative factors in predicting postoperative adverse outcomes in geriatric surgical patients. Am Geriatr Soc.

[2] Dunne JR, Malone D, Tracy JK, Gannon C, Napolitano LM (2002). Perioperative anemia: an independent risk factor for infection, mortality, and resource utilization in surgey. J Surg Res.

[3] Gruson KI, Aharonoff GB, Egol KA, Zuckerman JD, Koval KJ (2002). The relationship between admission hemoglobin level and outcome after hip fracture. J Orthop Trauma.

[4] Hardy JF, de Moerloose P, Samama CM (2006). Massive transfusion and coagulopathy: pathophysiology and implications for clinical management. Can J Anaesth.

[5] Harvey MP, Greenfield TP, Sugrue ME, Rosenfeld D (1995). Massive blood transfusion in a tertiary referral hospital. Clinical outcomes and haemostatic complications. Med J Aust.

[6] Bohlius J, Schmidlin K, Brillant C (2009). Recombinant human erythropoiesis-stimulating agents and mortality in patients with cancer: a meta-analysis of randomised trials. Lancet.

[7] Seghatchian J, Samama MM (2012). Massive transfusion: an overview of the main characteristics and potential risks associated with substances used for correction of a coagulopathy. Transfus Apher Sci.

[8] Ronnie AR, Stephen MK (2004). Assessment and management of the geriatric patient. Crit Care Med.

[9] Goodnough LT, Maniatis A, Earnshaw P (2010). Management of preoperative anaemia in patients undergoing elective surgery. ISBT Sci Ser.

[10] Johansson PI, Ostrowski SR, Secher NH (2010). Management of major blood loss: an update. Acta Anaesthesiol Scand.

[11] Lee A, Kerridge RK, Chui PT, Chiu CH, Gin T (2011). Perioperative systems as a quality model of perioperative medicine and surgical care. Health Policy.

[12] Heddle NM, Arnold DM, Boye D, Webert KE, Resz I, Dumont LJ (2008). Comparing the efficacy and safety of apheresis and whole blood-derived platelet transfusions: systematic review. Transfusion.

[13] Kuriyan M, Carson JL (2005). Anemia and clinical outcomes. Anesthesiol Clin North Am.

[14] Greenblatt DY, Rajamanickam V, Mell MW (2011). Predictors of surgical site infection after open lower extremity revascularization. J Vasc Surg.

[15] Carson JL, Terrin ML, Jay M (2003). Anemia and postoperative rehabilitation. Can J Anaesth.

[16] Giles KA, Hamdan AD, Pomposelli FB, Wyers MC, Siracuse JJ, Schermerhorn ML (2010). Body mass index: surgical site infections and mortality after lower extremity bypass from the National Surgical Quality Improvement Program 2005–2007. Ann Vasc Surg.

[17] O’Keeffe SD, Davenport DL, Minion DJ, Sorial EE, Endean ED, Xenos ES (2010). Blood transfusion is associated with increased morbidity and mortality after lower extremity revascularization. J Vasc Surg.

[18] Lindholm PF, Annen K, Ramsey G (2010). Approaches to minimize infection risk in blood banking and transfusion practice. Infect Disord Drug Targets.

[19] Cuenca J, Garcia-Erce JA, Martinez F, Cardona R, Pérez-Serrano L, Muñoz M (2007). Preoperative haematinics and transfusion protocol reduce the need for transfusion after total knee replacement. Int J Surg.

[20] Weiskopf RB, Kramer JH, Viele M (2000). Acute severe isovolemic anemia impairs cognitive function and memory in humans. Anesthesiology.

[21] Carson JL, Noveck H, Berlin JA, Gould SA (2002). Mortality and morbidity in patients with very low postoperative Hb levels who decline blood transfusion. Transfusion.

[22] Ferraris VA, Davenport DL, Saha SP, Austin PC, Zwischenberger JB (2012). Surgical outcomes and transfusion of minimal amounts of blood in the operating room. Arch Surg.

